# Never Give Alcohol in Cases of Stupor or Injuries

**Published:** 1910-03

**Authors:** T. D. Crothers

**Affiliations:** Hartford, Conn.


### Never Give Alcohol in Cases of Stupor or Injuries.

BY T. D. CROTHERS M. D., HARTFORD, CONN.

Modern research and experience shows that nothing is more dangerous than alcohol in any form given to a man with a crushed or injured leg, fractured bones, or when in a state of stupor from causes known or unknown.

Where the arteries, bones or flesh are crushed nature makes an effort to stop the bleeding by damming up the ruptured arteries and throwing out obstructive material to prevent the flow of blood. Where alcohol in any form is given the heart is roused and the current of blood rushes with greater velocity to all parts of the body, and where the arteries are ruptured, bleeding is greatly intensified. The more alcohol the greater the bleeding.

The very purpose of checking the blood current and restoring the life fluid is defeated. The blood-vessels that should relax and prevent the current from flowing are paralyzed and expanded. Take the ordinary fracture of bones. The broken parts are driven out and crushed into the flesh, tearing muscles and blood-vessels. By alcohol these crushed tissues are filled with blood which can not be removed and must hinder the healing process, and at all events increases inflammation, and the difficulty of replacing the bones. The man who falls down in a faint when on the street may have a slight rupture of some blood-vessel, and the slowing up of the heart is nature's effort to close this rupture and prevent it from growing wider, but alcohol given rouses up the heart, sends an increased current of blood, and the rupture is not only widened, but more blood driven through it, and perhaps other arteries in the neighborhood are cracked.

If one had a vulcanized rubber pipe that had become old and brittle, it would be very foolish to overcome a slight leak by sending an increased current of water through it. The leak would be made larger, and very likely other leaks would be made by the greater strain of an increased flow of water.

This same thing occurs in many instances where persons faint or become unconscious on the street, and are carried to a drug store or physician's office and given alcohol as the first aid and medicine. In heat or sun strokes the alcohol not only increases the flow of blood to the brain, but paralyzes the coats of the arteries and prevents the return of the blood.

The man picked up on the street, stupid, with an intensely red face, is suffering from some form of congestion. When given alcohol this is increased, and the greater redness of the face is the best indication of the actual condition present.

Sending more blood to the brain and paralyzing the vessels which return it is always more or less fatal. In injury, collapse and any kind of shock or derangement of the system, treated by alcohol for some supposed temporary effect, is always followed by a reaction and diminished vitality. The healing of wounds, the restoration of damaged circulation and the overcoming of congested states, and the closing of fractures and leaks in the blood-vessels are all impeded and made more difficult.

Nature's efforts to repair the injury are diminished and checked. It may be said with great certainty that no remody known in emergency cases is so dangerous as spirits in any form.

It may be also said that no remedy is more accessible, practical and safer than water applied in any way, either as a stream on the back of the neck or as bandages over the injured parts, or drunk in teaspoonful doses. If the slowness of the heart creates fear that it is going to stop altogether, water is the safest and best of all remedies, and alcohol is the very worst.

There is no reaction from water. There is no lowered vitality. No diminution of the power of repair. Instances occur in almost every city of injured and unconscious men who are given alcohol and who later die, and it is a most suggestive question how far alcohol was contributory to the death.

There are many cases in which this is very clear, and in all instances there is a strong probability that alcohol may have destroyed the powers of recovery. There is no theory in this, but it is hard common sense, which can be verified and proven, irrespective of all opinions.

